# Supplementary material for: A new strategy for durable control of late blight in potato by a single soil application of an oxathiapiprolin mixture in early season
Source: PLoS One. 2020 Aug 21;15(8):e0238148. doi: 10.1371/journal.pone.0238148 (PMC7444534; doi:10.1371/journal.pone.0238148)
Supplement: Attachment — Submitted filename: Response to Reviewrs and Editor Plos One 5.78.2020.docx [file pone.0238148.s001.docx]

5.8.2020

**Response to Editor and Reviewer 1**

The comment of the Editor was addressed: Reference list was reordered as he requested.

The comment of the Reviewer to spell Phytophthora infestans in italics was adopted.

Sincerely

Y Cohen

Professor
